# Tolerability, Safety, Pharmacokinetics and Drug Interaction of Cefotaxime Sodium–Tazobactam Sodium Injection (6:1) Following Single and Multiple Intravenous Doses in Chinese Healthy Subjects

**DOI:** 10.3389/fphar.2020.01033

**Published:** 2020-07-08

**Authors:** Ning Chen, Lu-Ning Sun, Wen-Hui Hu, Yi-Ya Wang, Li-Jun Xie, Juan Cheng, Hong-Wen Zhang, Yun Liu, Yong-Qing Wang, Li Ding

**Affiliations:** ^1^ Research Division of Clinical Pharmacology, First Affiliated Hospital of Nanjing Medical University, Nanjing, China; ^2^ Department of Pharmaceutical Analysis, China Pharmaceutical University, Nanjing, China; ^3^ Department of Bioanalysis, Nanjing Clinical Tech. Laboratories Inc., Nanjing, China

**Keywords:** cefotaxime, tazobactam, pharmacokinetics, safety, drug interaction

## Abstract

**Purpose:**

To evaluate the tolerability, safety, pharmacokinetics and drug interaction of cefotaxime sodium–tazobactam sodium injection (6:1) in Chinese healthy subjects. The results of the safety and pharmacokinetic studies supported further clinical trials.

**Method:**

A randomized, single-blind, ascending dose, placebo-controlled, single-center study was conducted. Sixty healthy subjects (38 males, 22 females) participated in this study. For the single-dose part, 0.47, 1.17, 2.34, 3.51, and 4.68 g of cefotaxime sodium–tazobactam sodium injection (6:1) was administered. For the multiple-dose part, the subjects were administered 2.34 and 3.51 g cefotaxime sodium–tazobactam sodium injection (6:1) three times a day for 7 consecutive days. For the drug interaction part, the subjects received 2.0 g cefotaxime sodium and 0.34 g tazobactam sodium alone and in combination.

**Results:**

Most adverse events and adverse drug reactions were mild. Moderate rash was considered a serious adverse event because of prolongation of hospitalization. The main pharmacokinetic parameters of cefotaxime and tazobactam had no significance difference between the 1.17, 2.34, and 3.51 g dose cohorts and between genders. There was no difference in trough concentrations on days 6, 7, and 8. The R*_C_*_max_ and R*_AUC_* were (0.921 ± 0.070) and (0.877 ± 0.057) for cefotaxime, and (0.913 ± 0.046) and (0.853 ± 0.060) for tazobactam, respectively. Following the administration of cefotaxime and tazobactam alone and in combination, the 90% conﬁdence intervals of the geometric mean ratios for *C*_max_ and *AUC* were within the predetermined range of 80–125%. In the single-dose part, the renal cumulative excretion ratios were (51.7 ± 6.2)% for cefotaxime, and (84.3 ± 8.1)% for tazobactam. There was no significant difference in the maximum excretion rates and cumulative excretion ratios for cefotaxime and tazobactam, alone or in combination.

**Conclusions:**

Cefotaxime sodium–tazobactam sodium injection (6:1) was well-tolerated at doses of 0.47 to 4.68 g. The pharmacokinetics of cefotaxime and tazobactam were reported as linear over a dose range of 1.17–3.51 g. Cefotaxime was partially excreted *via* urine, whereas tazobactam was mainly excreted *via* urine. There was no significant accumulation after administration over 7 consecutive days. The pharmacokinetics and excretion of cefotaxime and tazobactam were not affected by the co-administration of cefotaxime–tazobactam.

## Introduction

As a safe and low-toxicity antimicrobial, cefotaxime (CTX), which belongs to the third-generation cephalosporins is a prescribed antibiotic agent (Agüero et al., 1999; Barbarina et al., 2001; Scanes et al., 2001). It is a broad-spectrum antibiotic which remarkable activity against both Gram-positive and -negative bacteria (Koedijk et al., 2016; Béranger et al., 2017). CTX alone has been widely applied in clinical practice in the Chinese population (Agüero et al., 1999; Barbarina et al., 2001; Scanes et al., 2001). However, its efficacy has been seriously threatened by drug-resistant bacteria following wide clinical application (Consortti and Salgado, 2017; Lubna and Khan, 2017). However, the combination of a β-lactam antibiotic and a β-lactamase inhibitor has emerged to overcome this problem (Nguyen et al., 2014; Gao et al., 2016). Tazobactam (TAZ) is considered to have a stronger inhibitory effect on β-lactamases than first- and second-generation inhibitors (Papp-Wallace and Bonomo, 2016). The partner β-lactams, which can still play an antimicrobial role against a significant portion of extended-spectrum β-lactamases (ESBLs) (Gutiérrez-Gutiérrez et al., 2016), have been introduced into the clinical setting, such as piperacillin (PIP) and TAZ (Li et al., 2013; Chen et al., 2016; Murao et al., 2017), as well as ceftolozane (CLZ) and TAZ (Papp-Wallace and Bonomo, 2016; Kratzer et al., 2019). Cefotaxime sodium–tazobactam sodium (CTX–TAZ) (6:1) is a novel combination of β-lactam-β-lactamase inhibitor. β-lactamases can hydrolyze CTX, and TAZ can inactivate β-lactamase (Nguyen et al., 2014; Gao et al., 2016). CTX–TAZ (6:1) exerts synergistic antibacterial effect on drug-resistant bacteria. Compared with CTX–TAZ (1:1), (2:1), (3:1), and (4:1), this ratio reduces the amount of TAZ without affecting its efficacy, thereby reducing toxicity and cost. As CTX is used to treat urinary tract infections (Luthy et al., 1981), it is necessary to determine the pharmacokinetic (PK) profile not only in plasma but also in urine. The objective of this study was to explore the tolerability, safety, and PK profiles after single-dose and multiple-dose administrations of CTX–TAZ (6:1) in Chinese healthy subjects. This study also evaluated the urinary excretion and drug interaction of CTX–TAZ (6:1). Our PK analysis could pave the way for clinical approval and determining the appropriate dosing regimen of CTX–TAZ (6:1) injection.

### Subjects and Methods

#### Study Subjects

The study was conducted according to the principles of the current Declaration of Helsinki and Good Clinical Practice. It was approved by the institutional review board of the Ethics Committee of the First Affiliated Hospital of Nanjing Medical University prior to the study on January 13, 2017 (Approval Number: 2017-MD-014.A2). The drug clinical trial approval number was 2005L02508, as given by the China National Medical Products Administration.

Healthy subjects aged 18 to 45 years with body mass index (BMI) of 18.0–26.0 kg/m^2^ were included. They were deemed healthy based on a screening procedure that included clinical laboratory tests, 12-lead electrocardiogram (ECG), physical examinations and medical history. Sixty subjects without any significant cardiac, renal, gastrointestinal, hepatic, or metabolic conditions were eligible to participate in this study. All individual participants provided written informed consent before participating in any study-related procedures.

#### Study Design

This was a randomized, single-blind, ascending dose, placebo-controlled, single-center study, which was divided into three parts, a single-dose part, a multiple-dose part and a drug interaction part. Cefotaxime sodium–tazobactam sodium injection (6:1) (package: 1.17 g, bath no: 20160901), cefotaxime sodium injection (package: 1.0 g, bath no: 20160901) and tazobactam sodium injection (package: 0.17 g, bath no: 20160901) were provided by Nanjing Yoko Pharmaceutical Co., Ltd (Nanjing, China). Those were administered as a 1-h intravenous injection twice daily after being dissolved in 100 ml of 0.9% sodium chloride. The 0.9% sodium chloride injection was also used as a placebo.

**Figure 1:**
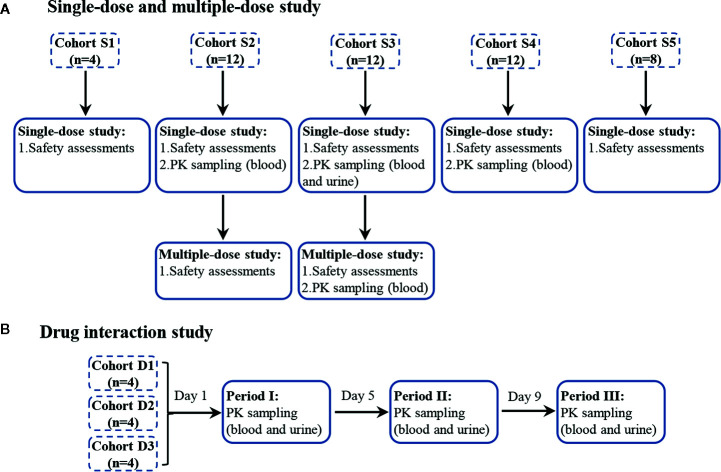
The progress of subjects through the trial, single-dose and multiple-dose part **(A)**, and drug interaction part **(B)**. Single-dose part: blood samples were collected at 0 h, 20 min, 40 min, 1 h, 1 h 15 min, 1 h 30 min, 1 h 45 min, 2 h, 2 h 30 min, 3 h, 3 h 30 min, 4 h, 5 h, 6 h, and 8 h. Urine samples were collected before dosing and in the period of 0–2, 2–5, 5–8, 8–12, and 12–16 h. Multiple-dose part: blood samples were collected at 0 h, 1 h on days 6, 7, 8, and at 0 h, 20 min, 40 min, 1 h, 1 h 15 min, 1 h 30 min, 1 h 45 min, 2 h, 2 h 30 min, 3 h, 3 h 30 min, 4 h, 5 h, 6 h, and 8 h on day 9. Drug interaction part: blood samples were collected at 0 h, 20 min, 40 min, 1 h, 1 h 15 min, 1 h 30 min, 1 h 45 min, 2 h, 2 h 30 min, 3 h, 3 h 30 min, 4 h, 5 h, 6 h, and 8 h. Urine samples were collected before dosing and in the period of 0–2, 2–5, 5–8, 8–12, and 12–16 h.

The flow diagram (**Figure 1**) was to illustrate the progress of subjects through the trial.

For the single-dose part, 48 subjects were randomly assigned to five dose cohorts and received intravenous infusion of 0.47 g (cefotaxime sodium 0.4 g, tazobactam sodium 0.07 g), 1.17 g (cefotaxime sodium 1.0 g, tazobactam sodium 0.17 g), 2.34 g (cefotaxime sodium 2.0 g, tazobactam sodium 0.34 g), 3.51 g (cefotaxime sodium 3.0 g, tazobactam sodium 0.51 g), or 4.68 g (cefotaxime sodium 4.0 g, tazobactam sodium 0.68 g) of CTX–TAZ (6:1) (**Table 1**). The next higher-dose cohort would be generated only after the safety and well-tolerability were confirmed in the previous dose cohort. Subjects received five different doses of CTX–TAZ (6:1) injection at 08:00 on the first day of study. Vital sign measurements, ECG, and safety assessments were performed at pre-dosing (0 h) and 1, 2, 4, and 8 h post-dosing. The urine-collected dose cohorts were banned from drinking water for 2 h after administration on the first day, and they should drink 200 ml of water at 2, 6, 8, 12, and 14 h after administration. The subjects of the 2.34 and 3.51 g dose cohorts continued to enroll in the multiple-dose part after completion of the single-dose part, administered CTX–TAZ (6:1) injection or a matching placebo three times a day for seven consecutive days (**Table 2**). CTX–TAZ (6:1) injection was administered at 08:00, 16:00, 00:00 from day 2 (day 1 of the multiple-dose part) to day 8 (day 7 of the multiple-dose part), and at 08:00 on day 9.

**Table 1:** Grouping for the single-dose part.

Cohort	Dose (g)	Research content	Number of subjects (drug/placebo)
S1	0.47	Tolerance, safety	4 (4/0)
S2	1.17	Tolerance, safety and pharmacokinetics	12 (10/2)
S3	2.34	Tolerance, safety, pharmacokinetics and urinary excretion	12 (10/2)
S4	3.51	Tolerance, safety and pharmacokinetics	12 (10/2)
S5	4.68	Tolerance, safety	8 (6/2)

**Table 2:** Grouping for the multiple-dose part.

Cohort	Dose (g)	Total daily dose (g)	Research content	Number of subjects (drug/placebo)
S3	2.34	7.02	Tolerance, safety, pharmacokinetics and urinary excretion	12 (10/2)
S4	3.51	10.53	Tolerance, safety	12 (10/2)

For the drug interaction part, three cohorts each received doses of 2.0 g of CTX, 0.34 g of TAZ, and 2.34 g of CTX–TAZ (6:1) in a three-period, three-sequence, and crossover design (**Table 3**). The procedure of the three-period study was the same as the single-dose part.

**Table 3:** Grouping for the drug interaction part.

Cohort	Number of subjects	Administration		
		Period I	Period II	Period III
D1	4	A (2.34 g CTX–TAZ)	B	C
D2	4	B (2.0 g CTX)	C	A
D3	4	C (0.34 g TAZ)	A	B

CTX, cefotaxime sodium injection; TAZ, tazobactam sodium injection; CTX–TAZ, Cefotaxime sodium–tazobactam sodium injection (6:1).

### Pharmacokinetic Sampling

For the single-dose PK study, venous blood samples (4 ml) were collected in sodium-heparinized tubes at pre-dosing (0 h) and 20 min, 40 min, 1 h, 1 h 15 min, 1 h 30 min, 1 h 45 min, 2 h, 2 h 30 min, 3 h, 3 h 30 min, 4 h, 5 h, 6 h, and 8 h post-dosing in the 1.17, 2.34, and 3.51 g dose cohorts. Urine samples were collected before dosing and in the period of 0–2, 2–5, 5–8, 8–12, and 12–16 h after dosing in the 2.34 g dose cohort. For the multiple-dose part, blood samples at before dosing and 1 h after dosing were collected on days 6, 7, and 8 in the 2.34 g dose cohort. In addition, multiple-dose PK sampling was also conducted at 0 h, 20 min, 40 min, 1 h, 1 h 15 min, 1 h 30 min, 1 h 45 min, 2 h, 2 h 30 min, 3 h, 3 h 30 min, 4 h, 5 h, 6 h, and 8 h after drug administration on day 9. For the drug interaction part, venous blood and urine samples were collected at the same time intervals as in the single-dose part. Following centrifugation at 3,500 rpm at 4°C for 8 min, plasma was separated and frozen at (−75 ± 10) °C until analysis. Urine samples were kept in a refrigerator at 4°C after collection. After each collection interval, the total volume was measured, and three aliquots of 1 ml were taken and stored in a refrigerator at (−75 ± 10) °C until analysis.

### Analytical Assays

Plasma and urine samples were analyzed by two validated HPLC-MS/MS methods using Shimadzu LC-30AD (Kyoto, Japan) and Applied Biosystems/Sciex Triple Quad™ 5500 (California, USA). The compounds were separated on an Ultimate XB-C18 column (50 × 2.1 mm, 3 μm; Welch) protected by a Security Guard Cartridges C18 (4 × 2.0 mm; Phenomenex) at 35°C. The solvents used for mobile phase consisted of methanol and solution A (0.05% acetic acid and 5 mM ammonium acetate). Mass spectrometry was performed using the negative electrospray ionization mode. Analytes were extracted from aliquots of 50.0 μl plasma or urine samples by protein precipitation with acetonitrile.

The standard curves were linear over concentration ranges of 0.150–150 μg/ml for CTX and 0.0200–10.0 μg/mL for TAZ in plasma, as well as 4.00–5,000 μg/ml for CTX and 0.300–500 μg/ml for TAZ in urine. The intra-day and interbatch precision and accuracy values well met the acceptance criteria according to the Guidance for Industry, Analytical Procedures and Methods Validation for Drugs and Biologics (2015) (U.S. Food and Drug Administration, 2015).

### Safety and Tolerability

For safety monitoring throughout the study, all subjects remained in the study unit under continuous observation. Details of adverse events (AEs) and adverse drug reactions (ADRs) were obtained and recorded by the study physicians. Clinical evaluation was required for subjects who have completed all trials, as well as subjects who withdrew from the trial. Routine safety and tolerability were evaluated through the basis of vital signs, physical examination, AEs, laboratory examination (routine blood, urine, and fecal test; occult blood test; and blood biochemical test), ECG, and the situation of withdrawal; this evaluation was performed at scheduled intervals during the studies. AEs that occurred during the study were classified as mild (awareness of a sign or symptom, but comfortably tolerated), moderate (discomfort that may interfere with daily activities), or serious (death, life-threatening, requiring hospitalization or incapacitating). AEs and ADRs were recorded and reported according to the Good Clinical Practice.

### Statistical Analysis

The sample size was set according to the guidance for clinical pharmacokinetic studies of chemical drugs, rather than the estimation of sample size. Descriptive statistics were used for safety and tolerability assessments. All AEs were analyzed together with the intensity and relationship to the medication and outcomes. The number and frequency of AEs and ADRs were calculated.

The PK parameters were assessed through noncompartmental analysis using Phoenix™ WinNonlin version 6.4. Statistical analysis was performed by Phoenix™ WinNonlin version 6.4 and SPSS version 19.0. In all cases, if the calculated P value was higher than 0.05, the difference would be considered statistically insignificant. For the single-dose PK study, one-way analysis of variance (ANOVA) and nonparametric test were used to compare PK parameters between the 1.17, 2.34, and 3.51 g dose cohorts. The linear relationships of PK parameters between the three dose cohorts were tested. An independent-sample *t*-test and a nonparametric test were applied to analyze the differences in PK parameters between males and females. Urinary drug excretion was analyzed by the key indicators, cumulative excretion ratio and maximum excretion rate. For the multiple-dose PK study, a one-way ANOVA was used to compare the trough concentrations on days 6, 7, and 8. The 95% confidence intervals (CIs) of the accumulation ratios, R*_C_*_max_ (*C*_ss_max_ of the multiple-dose part/*C*_max_ of the single-dose part), and *R_AUC_* (*AUC*_ss_ of the multiple-dose part/*AUC*_0–τ_ of the single-dose part), were estimated by Phoenix™ WinNonlin version 6.4. After administration of CTX and TAZ alone and in combination, the possibility of clinically meaningful drug interaction was evaluated to determine whether the 90% CIs of the geometric mean ratios for *C*_max_ and *AUC* were within the predetermined range of 80–125%. Paired-sample *t*-test was applied to compare the maximum excretion rates and the cumulative excretion ratios after CTX and TAZ administration alone and in combination.

## Results

### Study Population

Sixty healthy subjects completed the trial. One subject from the 2.34 g dose cohort withdrew on day 8. Other demographic data are provided in **Table 4**.

**Table 4:** Demographic characteristics of the subjects.

		Single-dose and multiple-dose part	Drug interaction part
Sex	Male	30	8
	Female	18	4
Age		26.4 ± 5.6	25.7 ± 3.6
BMI (kg/m^2^)		21.4 ± 2.1	22.8 ± 1.7

### Safety and Tolerability

In the single-dose part, eight AEs were reported in the 1.17 g dose cohort and one AE was reported in the 4.68 g dose cohort, while another subject treated with placebo reported three AEs. The incidence rates were 50.0% (2/4), 16.7% (1/6), and 12.5% (1/8), respectively. There was no ADR during this part. Those AEs were of mild intensity.

In the multiple-dose part, 4/10 (40.0%) subjects reported 12 AEs in the 2.34 g dose cohort and 2/10 (20.0%) subjects experienced five AEs in the 3.51 g dose cohort. In addition, 2/4 (50.0%) subjects, treated with placebo, reported two AEs. Five ADRs were reported by 3/10 (30.0%) subjects in the 2.34 g dose cohort and four ADRs were reported by 2/10 (20.0%) subjects in the 3.51 g dose cohort. Moreover, one ADR was reported in 1/4 (25.0%) subjects after placebo administration. Almost all AEs and ADRs reported in this part were of mild grade, with only two AEs reported as moderate (fever, rash). A subject had prolonged hospital stay due to moderate rash and discontinued the study on day 8 after multiple dosing in the 2.34 g dose cohort. Therefore, this AE was considered a serious AE (SAE) and determined to be probably related to the study medication.

### Single-Dose Pharmacokinetics and Urinary Excretion Study

**Figure 2:**
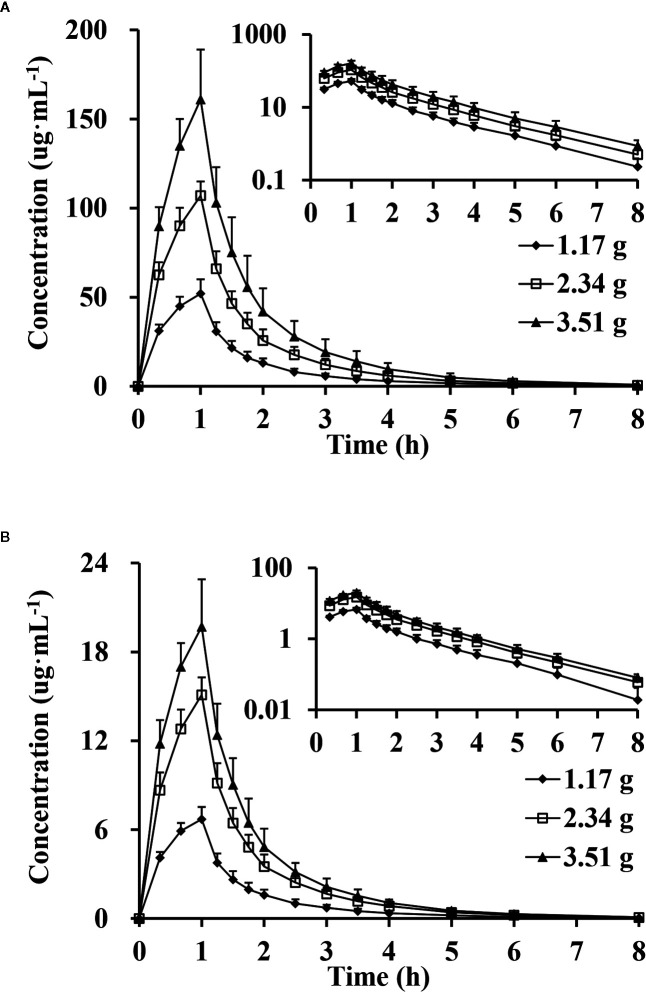
Mean plasma concentration versus time profiles, semi-log of mean plasma concentration versus time profiles after intravenous injection of doses of 1.17, 2.34 and 3.51 g of cefotaxime **(A)** and tazobactam **(B)** (n = 10).

**Figure 3:**
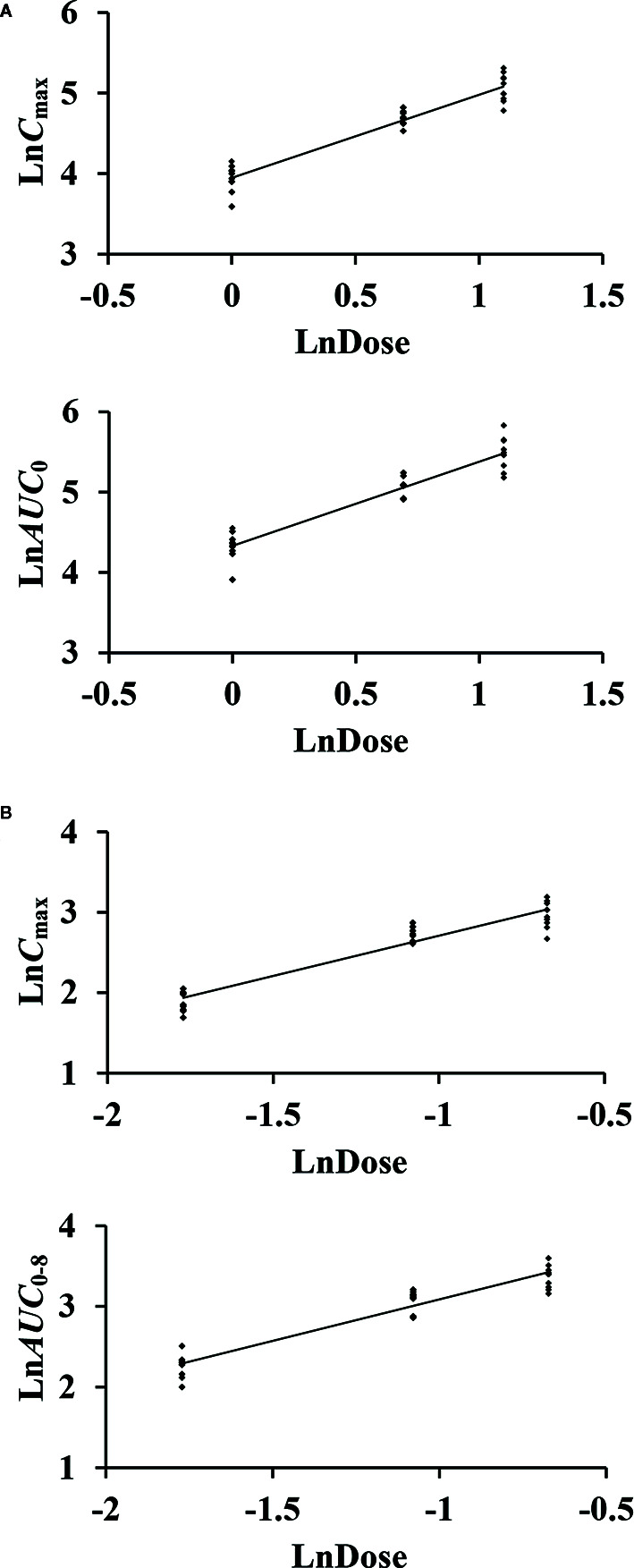
The relationship of the log-transformed *C*_max_ and *AUC*_0–8_ with the log-transformed doses of cefotaxime **(A)** and tazobactam **(B)**.

After single-dose administration, CTX and TAZ reached their *C*_max_ within 0.667–1.07 h. The PK parameters of the 1.17, 2.34, and 3.51 g dose cohorts are presented in **Table 5**. *T*_max_, *t*_1/2_, *MRT*_0–8_, *C*L, and *Vd* were not significantly different between the doses of 1.0, 2.0, 3.0 g of cefotaxime sodium and 0.17, 0.34, 0.51 g of tazobactam sodium (P >0.05). The comparison of PK parameters between male and female subjects is presented in **Table 6**. The results of independent-samples *t*-test and nonparametric test showed that those PK parameters, ln(*C*_max_/dose), ln(*AUC*_0–t_/dose), *t*_1/2_, *MRT*_0–8_, *C*L, *Vd*, and *T*_max_, had no significance difference between genders (P >0.05). The mean plasma concentrations versus time proﬁles of the analytes from the three dose cohorts are shown in **Figure 2**. One-way ANOVA revealed that the ln(*C*_max_/dose) and ln(*AUC*_0–8_/dose) of CTX and the ln(*AUC*_0–8_/dose) of TAZ were not significantly different between the three dose cohorts (P >0.05). The difference in the ln(*C*_max_/dose) of TAZ (P = 0.0190) may be due to the interindividual variability and the limited sample size. Therefore, it was not enough to regard this pharmacokinetics as nonlinear. The relationships of *C*_max_ and *AUC*_0–8_ with dose are shown in **Figure 3**. The slopes β (PK = α * Dose^β^) for the log-transformed *C*_max_ and *AUC*_0–8_ to the log-transformed doses were 1.03 and 1.05 of CTX, as well as 1.00 and 1.03 of TAZ, respectively (criteria range: 0.800–1.25). Therefore, this PK could be regarded as dose-proportional. The 90% CIs of the slope were contained within an interval of 0.797–1.20, which also indicated dose proportionality.

**Table 5:** PK parameters of cefotaxime and tazobactam after intravenous injection of single-dose and multiple-dose.

Parameter		1.17 g	2.34 g		3.51 g
		Single-dose(n = 10)	Single-dose(n = 10)	Multiple-dose(n = 9)	Single-dose(n = 10)
Cefotaxime	*C*_max_ (μg·ml^−1^)	52.0 ± 8.0	108 ± 9	99.8 ± 11.4	161 ± 28
	*T*_max_ (h)	1.00 ± 0.00	1.01 ± 0.02	0.963 ± 0.111	0.967 ± 0.105
	*K* (h^−1^)	0.640 ± 0.068	0.623 ± 0.066	0.658 ± 0.076	0.615 ± 0.058
	*t*_1/2_ (h)	1.09 ± 0.11	1.12 ± 0.11	1.07 ± 0.13	1.14 ± 0.11
	*CL* (L·h^−1^)	13.3 ± 2.6	12.7 ± 1.5	14.8 ± 1.4	12.7 ± 2.5
	*Vd* (L)	21.0 ± 4.6	20.5 ± 2.5	22.8 ± 2.8	20.8 ± 4.8
	*MRT*_0–8_ (h)	0.985 ± 0.134	0.995 ± 0.141	1.34 ± 0.11	1.03 ± 0.12
	*AUC*_0–8_ (μg·h·ml^−1^)	76.6 ± 12.3	159 ± 18	136 ± 13	244 ± 50
	*AUC*_0–∞_ (μg·h·ml^−1^)	77.1 ± 12.3	160 ± 18	136 ± 13	245± 50
	*C*_ss, av_ (μg·ml^−1^)	–	–	17.0 ± 1.6	–
	*DF*	–	–	5.85 ± 0.32	–
Tazobactam	*C*_max_ (μg·ml^−1^)	6.70 ± 0.82	15.4 ± 1.4	14.0 ± 1.4	19.8 ± 3.1
	*T*_max_ (h)	1.00 ± 0.00	1.01 ± 0.02	0.963 ± 0.111	0.967 ± 0.105
	*K* (h^−1^)	0.700 ± 0.090	0.658 ± 0.059	0.744 ± 0.092	0.662 ± 0.066
	*t*_1/2_ (h)	1.01 ± 0.12	1.06 ± 0.099	0.944 ± 0.111	1.06 ± 0.12
	*CL* (L·h^−1^)	17.7 ± 2.5	15.5 ± 2.0	18.4 ± 1.8	17.6 ± 2.4
	*Vd* (L)	25.6 ± 4.02	23.7 ± 2.5	24.9 ± 2.5	27.0 ± 6.0
	*MRT*_0–8_ (h)	0.938 ± 0.168	0.975 ± 0.145	1.33 ± 0.13	0.972 ± 0.088
	*AUC*_0–8_ (μg·h·ml^−1^)	9.70 ± 1.35	22.1 ± 2.5	18.6 ± 1.9	29.3 ± 4.1
	*AUC*_0–∞_ (μg·h·ml^−1^)	9.76 ± 1.33	22.2 ± 2.5	18.7 ± 1.9	29.5 ± 4.1
	*C*_ss, av_ (μg·ml^−1^)	–	–	2.33 ± 0.23	–
	*DF*	–	–	6.03 ± 0.46	–

**Table 6:** The comparison of PK parameters between male and female subjects.

Parameter		Male(n = 19)			Female(n = 11)		
		1.17 g	2.34 g	3.51 g	1.17 g	2.34 g	3.51 g
Cefotaxime	*C*_max_ (μg·ml^−1^)	51.9 ± 9.6	106 ± 10	159 ± 26	52.3 ± 3.4	112 ± 9	164 ± 33
	*T*_max_ (h)	1.00 ± 0.00	1.00 ± 0.00	0.945 ± 0.136	1.00 ± 0.00	1.02 ± 0.04	1.00 ± 0.00
	*K* (h^−1^)	0.633 ± 0.041	0.607 ± 0.071	0.627 ± 0.071	0.654 ± 0.124	0.647 ± 0.056	0.596 ± 0.028
	*t*_1/2_ (h)	1.10 ± 0.07	1.16 ± 0.13	1.12 ± 0.13	1.08 ± 0.20	1.08 ± 0.09	1.17 ± 0.05
	*CL* (L·h^−1^)	13.3 ± 3.1	12.5 ± 1.8	12.8 ± 2.5	13.3 ± 1.0	12.9 ± 1.1	12.4 ± 3.0
	*Vd* (L)	21.2 ± 5.5	20.7 ± 2.4	20.8 ± 4.8	20.7 ± 2.3	20.1 ± 3.0	20.9 ± 5.4
	*MRT*_0–8_ (h)	1.00 ± 0.12	1.07 ± 0.09	1.01 ± 0.09	0.942 ± 0.184	0.884 ± 0.130	1.06 ± 0.16
	*AUC*_0–8_ (μg·h·ml^−1^)	77.4 ± 14.7	161 ± 22	239 ± 42	74.7 ± 5.4	155 ± 12	252 ± 65
	*AUC*_0–∞_ (μg·h·ml^−1^)	77.9 ± 14.6	162 ± 22	240 ± 42	75.3 ± 5.6	156 ± 12	253 ± 66
Tazobactam	*C*_max_ (μg·ml^−1^)	6.71 ± 0.94	15.1 ± 1.1	19.9 ± 2.6	6.67 ± 0.63	15.8 ± 1.8	19.5 ± 4.2
	*T*_max_ (h)	1.00 ± 0.00	1.00 ± 0.00	0.945 ± 0.136	1.00 ± 0.00	1.02 ± 0.04	1.00 ± 0.00
	*K* (h^−1^)	0.697 ± 0.048	0.659 ± 0.069	0.663 ± 0.083	0.666 ± 0.125	0.655 ± 0.052	0.662 ± 0.036
	*t*_1/2_ (h)	0.999 ± 0.067	1.06 ± 0.11	1.06 ± 0.15	1.06 ± 0.18	1.06 ± 0.09	1.05 ± 0.06
	*CL* (L·h^−1^)	17.6 ± 3.0	15.4 ± 2.1	17.5 ± 2.1	18.0 ± 1.3	15.7 ± 2.2	17.8 ± 3.2
	*Vd* (L)	25.3 ± 4.4	23.4 ± 1.8	27.0 ± 6.4	27.3 ± 3.0	24.1 ± 3.6	27.1 ± 6.3
	*MRT*_0–8_ (h)	0.955 ± 0.160	1.03 ± 0.11	0.974 ± 0.069	0.911 ± 0.207	0.892 ± 0.163	0.970 ± 0.123
	*AUC*_0–8_ (μg h·ml^−1^)	9.83 ± 1.58	22.2 ± 2.5	29.4 ± 3.4	9.39 ± 0.62	21.9 ± 2.8	29.3 ± 5.6
	*AUC*_0–∞_ (μg·h·ml^−1^)	9.87 ± 1.57	22.3 ± 2.6	29.5 ± 3.4	9.48 ± 0.65	21.9 ± 2.8	29.4 ± 5.6

**Figure 4:**
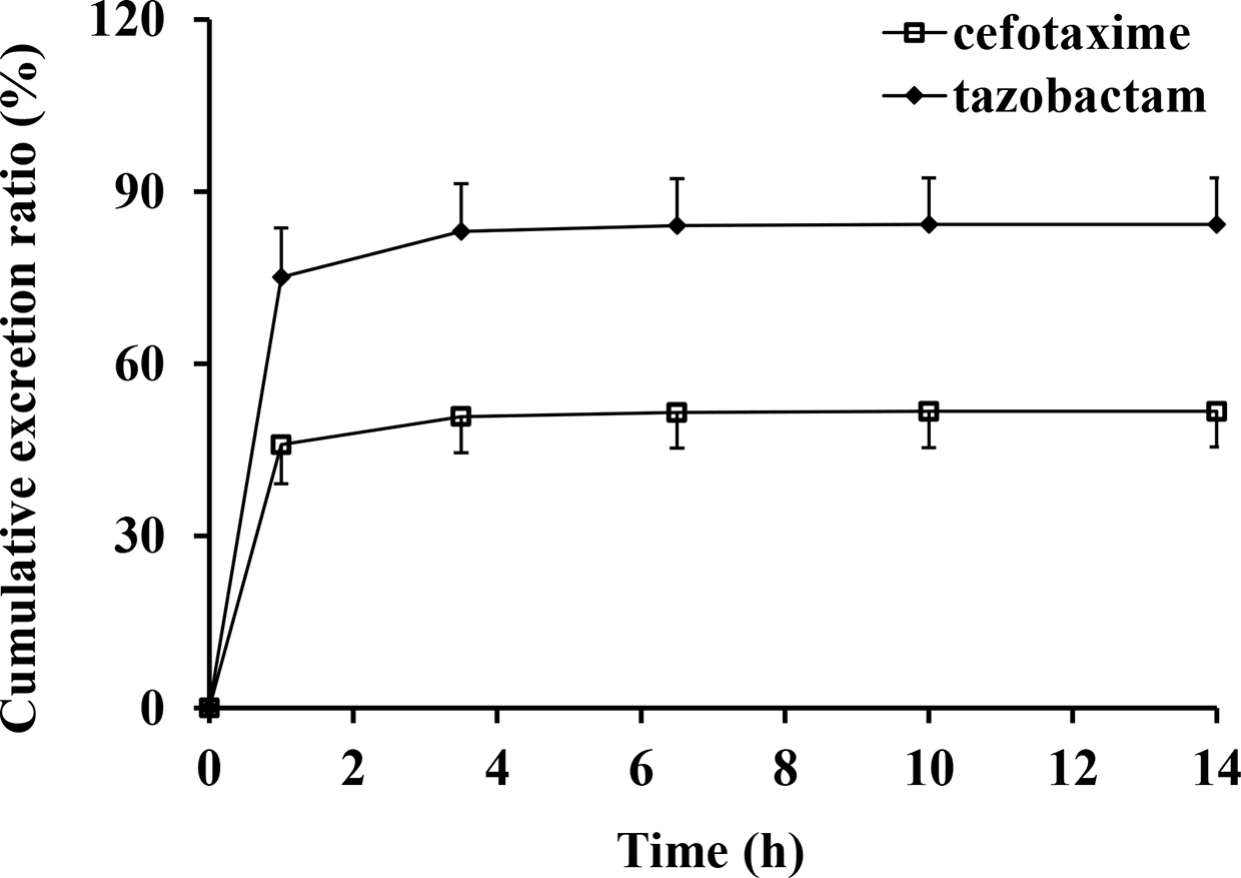
Mean cumulative excretion ratios versus time profiles and standard deviations after intravenous injection of cefotaxime and tazobactam (n = 10).

The excretion rates of CTX and TAZ in urine reached peak values at (0.459 ± 0.068) g/h of CTX and (0.128 ± 0.015) g/h of TAZ over 1 h in the 2.34 g dose cohort. The mean cumulative excretion ratios of CTX and TAZ are shown in **Figure 4**. The cumulative excretion ratios of CTX and TAZ were (51.7 ± 6.2) and (84.3 ± 8.1)%, respectively.

### Multiple-Dose Pharmacokinetics

The trough concentrations of the 2.34 g dose cohort on days 6, 7, and 8 were analyzed by one-way ANOVA. The results (P >0.05) showed no difference in the trough concentrations between days 6, 7, and 8. The plasma concentrations of CTX and TAZ all appeared to be at a steady state on day 6.

**Figure 5:**
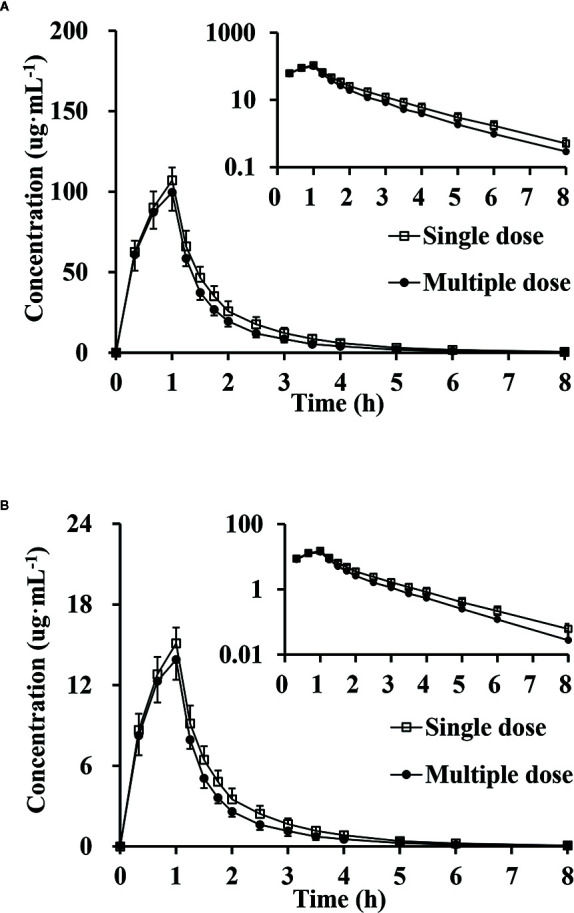
Mean plasma concentration, semi-log of mean plasma concentration-time profiles and standard deviations of cefotaxime **(A)** and tazobactam **(B)** after intravenous injection of single-dose and multiple-dose (n = 9).

The *R_C_*_max_ of CTX and TAZ were (0.921 ± 0.070) and (0.913 ± 0.046), the *R_AUC_* of CTX and TAZ were (0.877 ± 0.057) and (0.853 ± 0.060), respectively. The 95% CIs of CTX were 86.7–97.2% (*R_C_*_max_) and 82.9–92.2% (*R_AUC_*), and the 95% CIs of TAZ were 87.7–94.8% (*R_C_*_max_) and 80.6–89.6% (*R_AUC_*). The mean plasma concentration-time proﬁles on day 9 following the multiple-dose administration were similar to those on day 1 after the single-dose administration (**Figure 5**). Furthermore, the comparison of PK parameters between multiple-dose and single-dose administration is shown in **Table 5**. The PK parameters of CTX and TAZ were also similar on day 9 compared with those on day 1, suggesting little accumulation over the 7-day consecutive dosing period.

### Drug Interaction of Pharmacokinetics and Urinary Excretion Study

**Figure 6:**
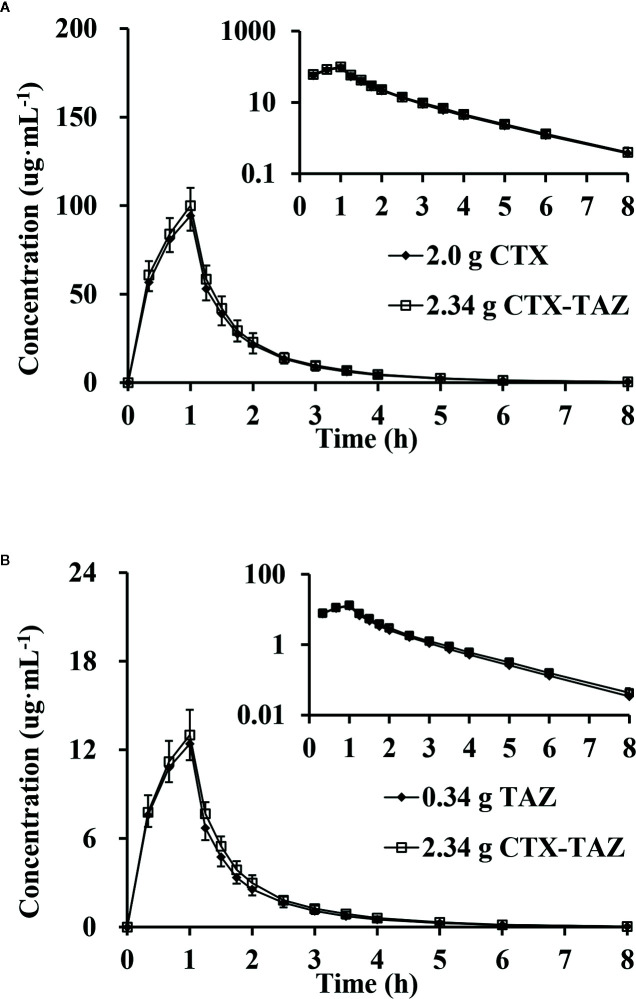
Mean plasma concentration, semi-log of mean plasma concentration-time profiles and standard deviations of cefotaxime **(A)** and tazobactam **(B)** after intravenous injection alone and in combination (n = 12). CTX, cefotaxime sodium injection; TAZ, tazobactam sodium injection; CTX–TAZ, Cefotaxime sodium–tazobactam sodium injection (6:1).

After administration of CTX and TAZ alone and in combination, the PK parameters of CTX and TAZ were measured, and the results are shown in **Table 7**. The concentration-time proﬁles of CTX and TAZ are shown in **Figure 6**. After CTX was administered alone and in combination with TAZ, the 90% CIs of the geometric mean ratios for *C*_max_, *AUC*_0–8_, and *AUC*_0–∞_ were 101.0–110.4%, 102.4–110.8% and 102.5–110.9%, respectively, within a predetermined range of 80–125%. After TAZ was administered alone and in combination with CTX, the 90% CIs of the geometric mean ratios for *C*_max_, *AUC*_0–8_ and *AUC*_0–∞_ were 98.9–110.3%, 104.1–113.8% and 104.0–113.7% within a predetermined range of 80–125%. These ﬁndings indicated that the PK parameters of both CTX and TAZ were unaffected by the co-administration.

**Table 7:** PK parameters of cefotaxime and tazobactam when given alone and in combination.

Parameter	Cefotaxime(n = 12)		Tazobactam(n = 12)	
	2.0 g CTX	2.34 g CTX–TAZ	0.34 g TAZ	2.34 g CTX–TAZ
*C*_max_ (μg·ml^−1^)	94.3 ± 8.5	99.6 ± 9.8	12.4 ± 1.1	13.0 ± 1.7
*T*_max_ (h)	1.00 ± 0.00	1.00 ± 0.00	0.972 ± 0.096	1.00 ± 0.00
*K* (h^−1^)	0.626 ± 0.054	0.619 ± 0.063	0.683 ± 0.085	0.688 ± 0.079
*t*_1/2_ (h)	1.12 ± 0.09	1.13 ± 0.12	1.03 ± 0.13	1.02 ± 0.12
*CL* (L·h^−1^)	15.2 ± 0.0	14.2 ± 0.0	20.1 ± 0.0	18.5 ± 0.0
*Vd* (L)	24.3 ± 0.0	23.1 ± 0.0	29.7 ± 0.0	27.1 ± 0.0
*MRT*_0–8_ (h)	0.915 ± 0.075	0.931 ± 0.081	0.869 ± 0.082	0.925 ± 0.063
*AUC*_0–8_ (μg h·L^−1^)	133 ± 16	142 ± 16	17.0 ± 1.5	18.5 ± 1.8
*AUC*_0–∞_ (μg·h·ml^−1^)	134 ± 16	143 ± 16	17.1 ± 1.5	18.6 ± 1.8

CTX, cefotaxime sodium injection; TAZ, tazobactam sodium injection; CTX–TAZ, Cefotaxime sodium–tazobactam sodium injection (6:1).

**Figure 7:**
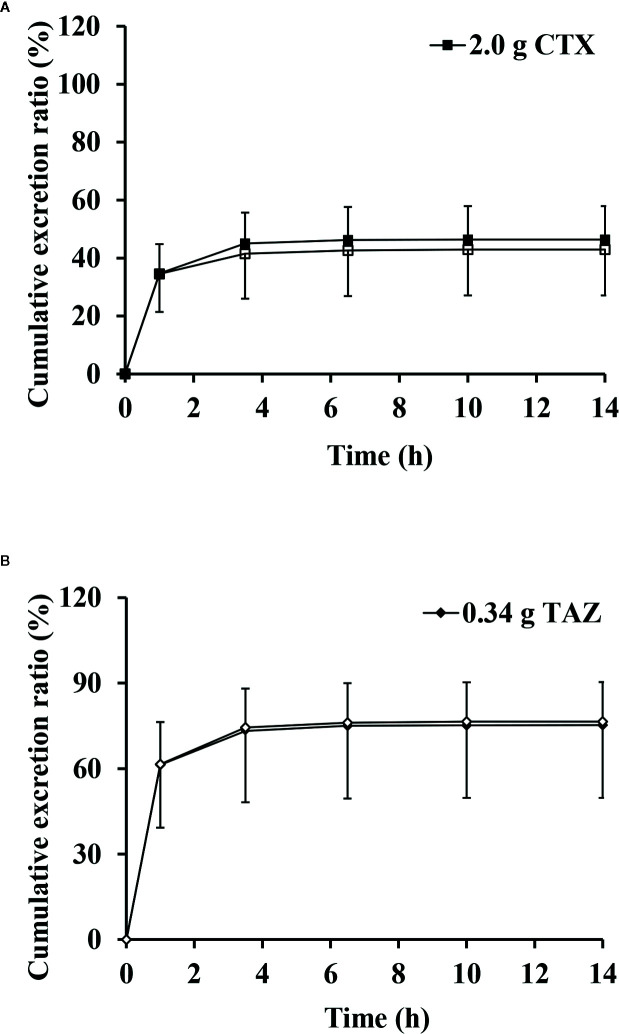
Mean cumulative excretion ratios versus time profiles and standard deviations of cefotaxime **(A)** and tazobactam **(B)** after intravenous injection alone and in combination (n = 12). CTX, cefotaxime sodium injection; TAZ, tazobactam sodium injection; CTX–TAZ, Cefotaxime sodium–tazobactam sodium injection (6:1).

The mean cumulative excretion ratios and standard deviations of CTX and TAZ when administered alone and in combination are shown in **Figure 7**. After administration of CTX alone, the maximum excretion rate and the cumulative excretion ratio were (0.384 ± 0.0586) g/h and (49.3 ± 7.1)%, respectively. When TAZ was administered alone, the maximum excretion rate and the cumulative excretion ratio of TAZ were (0.106 ± 0.0257) g/h and (75.8 ± 15.7)%, respectively. Similar results were observed when these two drugs were co-administered. After administration of CTX in combination with TAZ, the maximum excretion rate and the cumulative excretion ratio were (0.353 ± 0.128) g/h and (45.3 ± 14.5)% for CTX, and (0.105 ± 0.0396) g/h and (76.5 ± 26.8)% for TAZ. The results of the paired-sample *t*-test showed that there was no significant difference in the maximum excretion rates and cumulative excretion ratios for CTX and TAZ, both alone and in combination.

## Discussion

This study evaluated the PK profiles, safety and tolerability of CTX–TAZ (6:1) injection and the drug interaction of CTX and TAZ in Chinese healthy subjects.

Single doses of 0.47 to 4.68 g of CTX–TAZ (6:1) were generally safe and well-tolerated. Multiple doses of 2.34 to 3.51 g of CTX–TAZ (6:1) injection administered for seven consecutive days were also generally safe and well-tolerated. AEs were observed in all dose cohorts and placebo cohorts. Most AEs and ADRs were mild, only two AEs were reported as moderate. In this study, no dose-limiting toxicities occurred in single doses up to 4.68 g and multiple doses up to 3.51 g for seven consecutive days. Therefore, those AEs appeared to be dose-independent. However, an AE, moderate rash, occurred in the 2.34 g cohort in the multiple-dose part was reported as a SAE for prolonging hospital stay. The incidence of ADRs with CTX ranges from 1 to 10%, and rash is a common reaction (Babu and Sharmila, 2011). Babu (Babu and Sharmila, 2011) reported a case of a 7-day-old male neonate with CTX-induced near-fatal anaphylaxis. De Koning et al. (De Koning et al., 1983) reported that one patient developed erythematous macular rash 2 days after treatment with CTX.

The PK parameters *C*_max_ and *AUC* of CTX and TAZ were identiﬁed as linear over a dose range of 1.17–3.51 g of CTX–TAZ (6:1). In addition, semi-logarithmic graphs showed that both CTX and TAZ were linearly eliminated. In this study, we found that doses of 1.0, 2.0, and 3.0 g of cefotaxime sodium and 0.17, 0.34, and 0.51 g of tazobactam sodium had no effect on the PK parameters, *T*_max_, *t*_1/2_, *MRT*_0–8_, *C*L, and *Vd*. Those findings in the present study were similar with previously published results. Xu et al. (2005) reported that there was no significant difference in *t*_1/2_ between doses of 0.9, 1.8, and 4.5 g of cefotaxime sodium and 0.3, 0.6, and 1.5 g of tazobactam sodium. Moreover, we found that genders did not lead to significant differences in the PK behavior. The plasma concentrations of CTX and TAZ all appeared to be at a steady state on day 6 with no meaningful accumulation of CTX and TAZ after multiple dosing. Those results supported further clinical trial and clinical application of CTX–TAZ (6:1) injection.

After CTX and TAZ were administered alone and in combination, the 90% CIs of the geometric mean ratios for *C*_max_ and *AUC* were within the predetermined range of 80–125%. This result indicated that the *C*_max_ and *AUC* of both CTX and TAZ were unaffected by the co-administration. Furthermore, **Table 7** showed that the PK parameters, *t*_1/2_ *, T*_max_, *K*, *CL*, *Vd* and *MRT*_0–8_ were not affected by the co-administration of CTX and TAZ. Several reports have described the PK profiles and safety of CTX and TAZ individually or in combination with other drugs (Luthy et al., 1981; Hary et al., 1989; Wise et al., 1991; Urien et al., 2004; Miller et al., 2012). Luthy et al. (1981) reported that the *t*_1/2_ of CTX was (0.72 ± 0.30) h in six healthy males after administration of CTX. Ings et al. (1985) reported that the *t*_1/2_ of CTX varied between 0.92 and 1.65 h in three healthy males after intravenous dosing of CTX. Hary et al. (1989) reported that the *t*_1/2_ of CTX was (1.59 ± 0.57) h in eight healthy subjects after administration of CTX. However, the sample size of those studies was small. Therefore, the main reasons for this difference might be individual differences. Wise et al. (1991) reported that the *t*_1/2_ of TAZ was (1.13 ± 0.21) h in six subjects after a single intravenous injection of 0.5 g TAZ. Miller et al. (2012) reported that the average *t*_1/2_ of TAZ was 0.970 and 1.10 h after single and multiple intravenous injection of TAZ, respectively. Those results also suggested that *t*_1/2_ was not affected by the co-administration of CTX–TAZ.

In the single-dose part, the renal cumulative excretion ratios were (51.7 ± 6.2)% for CTX, and (84.3 ± 8.1)% for TAZ. Those data agreed with a previous study evaluating urinary excretion following co-administration of CTX–TAZ (3:1) (Xu et al., 2005). After administration of CTX alone, the cumulative excretion ratio was (49.3 ± 7.1)%. When TAZ was administered alone, the cumulative excretion ratio of TAZ was (75.8 ± 15.7)%. After co-administration, the cumulative excretion ratios were (45.3 ± 14.5)% for CTX and (76.5 ± 26.8)% for TAZ. Ings et al. (1985) reported that the urinary excretion of CTX was approximately 57%, and Luthy et al. (1981) reported that the urinary excretion was (53 ± 6)% after the administration of CTX alone. The cumulative excretion ratio of CTX after co-administration was in contrast to the cumulative excretion ratio of CTX administered alone in a previous research, which also suggested TAZ had no effect on the excretion of CTX. However, Wise et al. (1991) reported that the mean cumulative excretion ratio of TAZ was (63.7 ± 7.9)% when it was administered alone. However, the previous study obtained data from only six healthy males. The renal cumulative excretion ratios were also different between the single-dose part and the drug interaction part in this study. Therefore, the main reasons for this difference might be individual differences. Furthermore, the results obtained from patients were different, which was probably related to variability of the liver and renal function between healthy subjects and patients (Hary et al., 1989; Urien et al., 2004). Therefore, follow-up studies should include PK evaluation to develop more appropriate dosing regimens for patients.

## Conclusions

Overall, CTX–TAZ (6:1) injection was a novel β-lactam-β-lactamase inhibitor combination with linear pharmacokinetics over a range of 1.17–3.51 g (1.0–3.0 g for cefotaxime sodium, 0.17–0.51 g for tazobactam sodium). It was also well-tolerated at doses of 0.47 to 4.68 g in Chinese healthy subjects. CTX and TAZ were rapidly cleared with a short *t*_1/2_. The doses of 1.0, 2.0, and 3.0 g for cefotaxime sodium and 0.17, 0.34, and 0.51 g for tazobactam sodium, as well as genders, did not lead to significant differences in the PK parameters. CTX was partially excreted *via* urine and TAZ was mainly excreted *via* urine. There was no significant accumulation after administration over 7 consecutive days in healthy subjects. The peak concentration, *in vivo* exposure, and the renal excretion were not affected by the co-administration of CTX and TAZ.

## Data Availability Statement

The data that support the findings of this study are available from the corresponding author upon reasonable request.

## Ethics Statement

The studies involving human participants were reviewed and approved by the institutional review board of Ethics Committee of the First Affiliated Hospital of Nanjing Medical University. The patients/participants provided their written informed consent to participate in this study.

## Author Contributions

NC wrote the manuscript. NC, L-NS, W-HH, and Y-YW participated in the method development, method validation, sample analysis, and statistical analysis, L-NS, L-JX, JC, H-WZ, YL, and Y-QW participated in the clinical research. L-NS, Y-QW, and LD participated in the research design. All authors contributed to the article and approved the submitted version.

## Funding

This project was supported by the Priority Academic Program Development of Jiangsu Higher Education Institutions, National Natural Sciences Foundation of China (81673515, 81503160, 81870436), Natural Science Foundation of Jiangsu Province (BK20161591), National Science and Technology Major Project (2018ZX09734007).

## Conflict of Interest

W-HH was employed by Nanjing Clinical Tech. Laboratories Inc.

The remaining authors declare that the research was conducted in the absence of any commercial or financial relationships that could be construed as a potential conflict of interest.

## References

[B1] Agüero J. Peris José-Esteban San-Martín E. (1999). Validation of a high-performance chromatographic method for determination of cefotaxime in biological samples. Fresenius J. Analyt. Chem. 363 (3), 289–293. 10.1007/s002160051190

[B2] Béranger A. Oualha M. Urien S. Genuini M. Renolleau S. Aboura R. (2017). Population pharmacokinetic model to optimize cefotaxime dosing regimen in critically ill children. Clin. Pharmacokinet. 57 (9), 1–9. 10.1007/s40262-017-0602-9 28980166

[B3] Babu T. A. Sharmila V. (2011). Cefotaxime-induced near-fatal anaphylaxis in a neonate: A case report and review of literature. Indian J. Pharmacol. 43 (5), 611–612. 10.4103/0253-7613.84987 22022015 PMC3195142

[B4] Barbarina N. Tilquin B. Hoffmann E. D. (2001). Radiosterilization of cefotaxime: investigation of potential degradation compounds by liquid chromatography-electrospray mass spectrometry. J. Chromatography A. 929 (1), 51–61. 10.1016/S0021-9673(01)01175-X 11594403

[B5] Chen Y. W. Lu J. M. Dong M. Wu D. Zhu Y. Q. Li Q. (2016). Target attainment analysis and optimal sampling designs for population pharmacokinetic study on piperacillin/tazobactam in neonates and young infants. Eur. J. Clin. Pharmacol. 72 (12), 1479–1488. 10.1007/s00228-016-2131-0 27644691

[B6] Consortti LíviaP. Salgado HéridaR. N. (2017). A critical review of analytical methods for quantification of cefotaxime. Crit. Rev. Analyt. Chem. 47 (4), 359–371. 10.1080/10408347.2017.1298988 28287269

[B7] De Koning G. A. Van Tio D. d H. J. A. Van Klingeren B. (1983). Single 1 g dose of cefotaxime in the treatment of infections due to penicillinase-producing strains of neisseria gonorrhoeae. Sexually Transmitted Infections 59 (2), 100–102. 10.1136/sti.59.2.100 PMC1046147 6299449

[B8] Gao C. L. Tong J. Yu K. J. Sun Z. D. An R. Du Z. M. (2016). Pharmacokinetics of cefoperazone/sulbactam in critically ill patients receiving continuous venovenous hemofiltration. Eur. J. Clin. Pharmacol. 72 (7), 823–830. 10.1007/s00228-016-2045-x 27023465

[B9] Gutiérrez-Gutiérrez Belén Pérez-Galera S. Elena S. Marina de C. Esther C. Benito A. (2016). A Multinational, Preregistered Cohort Study of β-Lactam/β-Lactamase Inhibitor Combinations for Treatment of Bloodstream Infections Due to Extended-Spectrum-β-Lactamase-Producing Enterobacteriaceae. Antimicrobial Agents Chemother. 60 (7), 4159–4160. 10.1128/AAC.00365-16 PMC4914653 27139473

[B10] Hary L. Andrejak M. Leleu S. Orfila J. Capron J. P. (1989). The pharmacokinetics of ceftriaxone and cefotaxime in cirrhotic patients with ascites. Eur. J. Clin. Pharmacol. 36 (6), 613–616. 10.1007/bf00637745 2776819

[B11] Ings R. M. J. Reeves D. S. White L. O. Bax R. P. Bywater M. J. Holt H. A. (1985). The human pharmacokinetics of cefotaxime and its metabolites and the role of renal tubular secretion on their elimination. J. Pharmacokinet. Biopharmaceut. 13 (2), 121–142. 10.1007/bf01059394 4057054

[B12] Koedijk J. B. Valkswinkels C. G. H. Rijpstra T. A. Touw D. J. Mulder P. G. H. Voort P. H. J. V. D. (2016). Pilot study of the pharmacokinetics of cefotaxime in critically ill patients with acute kidney injury treated with continuous renal replacement therapy. Antimicrob. Agents Chemother. 60 (6), 3587–3590. 10.1128/AAC.02888-15 27021325 PMC4879359

[B13] Kratzer A. Schießer S. Matzneller P. Wulkersdorfer B. Zeitlinger M. Schlossmannd J. (2019). Determination of total and free ceftolozane and tazobactam in human plasma and interstitial fluid by HPLC-UV. J. Pharmaceut. Biomed. Anal. 163, 34–38. 10.1016/j.jpba.2018.09.044 30278324

[B14] Li Z. P. Chen Y. W. Li Q. Di C. Shi W. J. Cao Y. (2013). Population pharmacokinetics of piperacillin/tazobactam in neonates and young infants. Eur. J. Clin. Pharmacol. 69 (6), 1223–1233. 10.1007/s00228-012-1413-4 23354809

[B15] Lubna M. Khan A. U. (2017). Synergistic effect of doripenem and cefotaxime to inhibit ctx-m-15 type β-lactamases: biophysical and microbiological views. Front. Pharmacol. 8, 449. 10.3389/fphar.2017.00449 28725196 PMC5496960

[B16] Luthy R. Blaser J. Bonetti A. Simmen H. Wise R. Siegenthaler W. (1981). Comparative multiple-dose pharmacokinetics of cefotaxime, moxalactam, and ceftazidime. Antimicrobial Agents Chemother. 20 (5), 567–575. 10.1128/AAC.20.5.567 PMC181752 6275776

[B17] Miller B. Hershberger E. Benziger D. Trinh M. Friedland I. (2012). Pharmacokinetics and safety of intravenous ceftolozane-tazobactam in healthy adult subjects following single and multiple ascending doses. Antimicrob. Agents Chemother. 56 (6), 3086–3091. 10.1128/AAC.06349-11 22450972 PMC3370713

[B18] Murao N. Ohge H. Ikawa K. Watadani Y. Uegami S. Shigemoto N. (2017). Pharmacokinetics of piperacillin-tazobactam in plasma, peritoneal fluid and peritoneum of surgery patients, and dosing considerations based on site-specific pharmacodynamic target attainment. Int. J. Antimicrobial Agents 50, 393–398. 10.1016/j.ijantimicag.2017.03.025 28694230

[B19] Nguyen H. M. Shie R. K. L. Graber C. J. (2014). Determining a clinical framework for use of cefepime and β-lactam/β-lactamase inhibitors in the treatment of infections caused by extended-spectrum-β-lactamase-producing enterobacteriaceae. J. Antimicrobial Chemother. 69 (4), 871–880. 10.1093/jac/dkt450 24265230

[B20] Papp-Wallace K. M. Bonomo R. A. (2016). New β-lactamase inhibitors in the clinic. Infect. Dis. Clinics North America 30 (2), 441–464. 10.1016/j.idc.2016.02.007 PMC4980828 27208767

[B21] Scanes T. Hundt A. F. Swart K. J. Hundt H. K. (2001). Simultaneous determination of cefotaxime and desacetylcefotaxime in human plasma and cerebrospinal fluid by high-performance liquid chromatography. J. Chromatography B. Biomed. Sci. App. 750 (1), 171–176. 10.1016/S0378-4347(00)00417-5 11204218

[B22] U.S. Food and Drug Administration (2015). Guidance for Industry, Analytical Procedures and Methods Validation for Drugs and Biologics. http://www.fda.gov/BiologicsBloodVaccines/GuidanceComplianceRegulatoryInformation/Guidances/default.htm Accessed 24 July 2015.

[B23] Urien S. Laurent N. Barre J. Druguet M. Bouvier D’yvoire M. Maire P. (2004). Pharmacokinetic modelling of cefotaxime and desacetylcefotaxime—a population study in 25 elderly patients. Eur. J. Clin. Pharmacol. 60 (1), 11–16. 10.1007/s00228-003-0725-9 14767629

[B24] Wise R. Logan M. Cooper M. Andrews J. M. (1991). Pharmacokinetics and tissue penetration of tazobactam administered alone and with piperacillin. Antimicrobial Agents Chemother. 35 (6), 1081–1084. 10.1128/AAC.35.6.1081 PMC284290 1656853

[B25] Xu L. Sheng Y. C. Zheng Q. S. (2005). Study on Pharmacokinetics and algorithm of cefotaxime sodium tazobactam sodium for intravenous drip in healthy volunteers. Chin. J. Clin. Pharmacol. Ther. 10 (11), 1225–1230. 10.3969/j.issn.1009-2501.2005.11.005

